# Cumulative IT Use Is Associated with Psychosocial Stress Factors and Musculoskeletal Symptoms

**DOI:** 10.3390/ijerph14121541

**Published:** 2017-12-08

**Authors:** Billy C. L. So, Andy S. K. Cheng, Grace P. Y. Szeto

**Affiliations:** Department of Rehabilitation Sciences, The Hong Kong Polytechnic University, Hong Kong, China; andy.cheng@polyu.edu.hk (A.S.K.C.); grace.szeto@polyu.edu.hk (G.P.Y.S.)

**Keywords:** ergonomics, mobile device, touchscreen, psychosocial, musculoskeletal disorders

## Abstract

This study aimed to examine the relationship between cumulative use of electronic devices and musculoskeletal symptoms. Smartphones and tablet computers are very popular and people may own or operate several devices at the same time. High prevalence rates of musculoskeletal symptoms associated with intensive computer use have been reported. However, research focusing on mobile devices is only just emerging in recent years. In this study, 285 persons participated including 140 males and 145 females (age range 18–50). The survey consisted of self-reported estimation of daily information technology (IT) exposure hours, tasks performed, psychosocial stress factors and relationship to musculoskeletal discomfort in the past 12 months. Total IT exposure time was an average of 7.38 h (±5.2) per day. The psychosocial factor of “working through pain” showed the most significant association with odds ratio (OR) ranging from 1.078 (95% CI = 1.021–1.138) for elbow discomfort, to 1.111 (95% CI = 1.046–1.180) for shoulder discomfort. Desktop time was also significantly associated with wrist/hand discomfort (OR = 1.103). These findings indicate only a modest relationship but one that is statistically significant with accounting for confounders. It is anticipated that prevalence rates of musculoskeletal disorders would rise in the future with increasing contribution due to psychosocial stress factors.

## 1. Introduction

Computer use at work is associated with increased risk for musculoskeletal disorders due to the static posture adopted and the highly repetitive hand motions [1,2]. In recent years, people not only use computers at work or at school, they are also using mobile phones, laptop or tablet computers for communication, leisure and entertainment. In particular, the touchscreen products such as multi-touch smartphones and tablet computers have become a major fascination around the world. In Asian countries, this phenomenon seems to be even more overwhelming, as the number of smartphone users is expected to top 347 million by 2015 [3]. Hong Kong is among one of the top selling markets in smartphone technology in the world, with an impressive 48% smartphone penetration rate in 2011 which is double the average purchase rate of smartphones around the world [4].

Past research has shown that computer use for over four hours per day would contribute to increased risk for musculoskeletal disorders (MSD) [1,2]. These disorders include neck pain, shoulder strain, forearm tenosynovitis, carpal tunnel syndrome, and de Quervain’s syndrome [5]. For mobile phone use, excessive neck flexion posture and thumb joint strain have been reported to be contributing factors to musculoskeletal disorders and these studies were based on conventional keypad phones [6,7]. So far, only a few laboratory studies have examined the physical demands in using multi-touch devices [8,9,10,11], reporting on the neck and upper limb posture and muscle activity in using smartphone devices. These are mainly based on standardized tasks of short durations from 1 to 10 min in either sitting or standing positions. A recent systematic review has reported that there is some evidence for the factors of neck flexion, frequency of phone calls, texting and gaming contributing to musculoskeletal complaints among mobile device users [12].

The etiology of musculoskeletal disorders is still not completely understood, but there is general consensus that these disorders are multifactorial in nature [13]. Both the physical exposures at work and psychosocial risk factors have been reported to have strong associations with neck, shoulder and forearm/hands complaints. Physical exposure factors can be related to static neck and arm postures, repetitive tasks, and workplace design [14,15,16]. Prior research indicates that a habitual pattern of increased muscle activity in the neck and shoulder region is a common phenomenon among office workers with chronic neck and shoulder pain [17]. Psychosocial factors related to job characteristics, high job demands, lack of job autonomy, and limited support from coworkers or supervisors have also been reported to be common factors associated with musculoskeletal discomforts in office workers [16,18].

A recent study on a large sample of IT workers in India (*n* = 4500) has reported a high correlation of “workstyle” scores with musculoskeletal pain, discomfort and loss of productivity [19]. The concept of Workstyle was introduced by Feuerstein and colleagues, as a “behavioural, cognitive and physiological response that can occur in individuals to increased work demands” [20,21]. The Workstyle model hypothesizes that how a worker performs his/her work is the result of the combined influence of psychological, physiological and behavioural responses to high work demands. The model proposes that these potentially high-risk behaviours may be driven by perceived stress and negative cognitions about work demands and discomforts. How this may affect the current generation of multiple device users is one of the research questions in this study.

While multi-touch devices are lightweight and easy to use, long durations of use and highly repetitive finger actions may impose significant strains on the musculoskeletal system. The primary objective of the present study was to find out the average duration of use of the various electronic devices among the young working population in Hong Kong. The secondary objective was to investigate whether cumulative exposure to IT devices would contribute to musculoskeletal discomforts among these IT device users. Besides the duration of IT use, physical and psychosocial factors at work may also contribute to the musculoskeletal symptoms, and this association is also examined in the present study.

## 2. Materials and Methods

### 2.1. Study Design and Participants

This study employed a cross-sectional design with a paper-based questionnaire and convenience sampling. The subjects were recruited from the local university and nearby physiotherapy clinics. Altogether, 285 persons completed the questionnaire survey out of 437 questionnaires distributed yielding a response rate of 65.2%. Young people who were intensive users of computers and mobile phones were the target group. To be included, each person had to use computers and/or mobile phone for at least 1–2 h per day. Participants were excluded if they suffered from chronic diseases affecting the musculoskeletal system such as rheumatoid arthritis, osteoarthritis and other connective tissue disorders, or if they had previous trauma or surgery in the upper extremity or spine. Prior to the study, ethical approval was obtained from the local university. Participants had to read the study information sheet and sign the informed consent form before proceeding to complete the questionnaire.

### 2.2. Questionnaire Design

The questionnaire consisted of the following parts: (i) personal information including age, gender, body height and weight, occupation, (ii) profile of use of various forms of IT—including desktop computers, laptops, mobile phones (keypad and multi-touch), tablet computers and handheld electronic game devices, (iii) subjective musculoskeletal discomfort in past 12 months (average) in neck and upper limb regions on a numerical rating scale (0–10), and (iv) the Short Form Workstyle. The profile of IT device use included factors such as duration of use of each device. For the multi-touch devices, additional information included location of use, type of task performed, input method and the posture adopted.

The Workstyle questions were presented in a bilingual mode with both English and Chinese text for each question. The Chinese version of Workstyle Short Form was validated in a previous study [22] that reported a high internal consistency (α = 0.84) and a good test-retest (3 weeks) reliability (r = 0.79 to 0.91). The Workstyle Short Form also has a well-established scoring system, with eight sub-scales including working through pain, social reactivity, limited workplace support, deadlines, self-imposed workspace, breaks, mood and autonomic response [23,24].

### 2.3. Data Analysis

The personal characteristics of the participants and their employment information were first presented as descriptive statistics. In the questionnaire, the participants were asked to indicate their daily hours of use of various types of IT devices. Those who reported <1 h was considered as “0”, 1–2 h as “1”, 2–4 h as “3”, 4–6 h as “5”, 6–8 h as “7”, and >8 h as “9”. These values were summed to provide an estimate of total IT time. Information was obtained about how the multi-touch devices were used—such as the venue, the tasks performed, and postures adopted. These data are summarized in Table 3. The characteristics of musculoskeletal symptoms in the neck, upper back and upper limb regions are also presented in descriptive statistics.

Altogether 16 independent variables were identified which included daily use time of 6 types of IT devices, total IT time, 8 subscale scores in workstyle, and the total workstyle score. Logistic regression models were built for each of the musculoskeletal symptom body region (neck, upper back, shoulder, elbow, wrist/hand). Univariate analysis was performed first with each independent variable and only those variables that reached *p* < 0.1 were entered into the multivariate regression model. Finally, multivariate stepwise (backward) logistic regression models were constructed for the discomfort in the 5 body regions. SPSS 20.0 (SPSS Inc., Chicago, IL, USA) was used for all data analysis and a significance value of *p* < 0.05 was adopted in all analyses.

## 3. Results

Data from 285 individuals were obtained. Most of the respondents (77%) were employed on a full-time basis with a small percentage being full-time students (see Table 1). Their demographic profiles showed that these were mostly in the younger age groups with 76% being in the age range of 18–40, and the remaining 24% were in the age range from 41 to over 50 years old.

### 3.1. Cumulative Exposure to IT Devices

Table 2 is a summary of the self-reported daily use patterns of various IT devices among the respondents. The majority used a desktop computer daily (78.9%), and the number of persons using multi-touch mobile phones is higher (89.8%). Among the 256 smartphone users, 55% reported 1–4 h of use daily while 25% reported four to over eight hours of use. The remaining 20% may be users of mobile phones of the traditional keypad style. However, we cannot be totally certain of this, as participants were allowed to make multiple responses to this question. The use of touchscreen or “multi-touch” devices was also further analysed and the results are presented in Table 3. The number of multi-touch smartphone users far exceeded that of the tablet devices. Among the respondents, 89 persons reported using two multi-touch smartphone devices on a regular basis. Nevertheless, it is worth noting that a very high percentage of the respondents used the smartphones and the tablets for browsing the internet, sending and receiving text messages, playing games, taking photos, and listening to music (all with >50% response rates from participants). The postures associated with use of handheld devices generally involved viewing the screen at below the eye level and forearms in unsupported positions.

When the daily reported hours of the various IT devices were added up, the results showed a mean value of 7.38 h ± 5.20 for total IT time.

### 3.2. Musculoskeletal Discomfort

**Figure 1:**
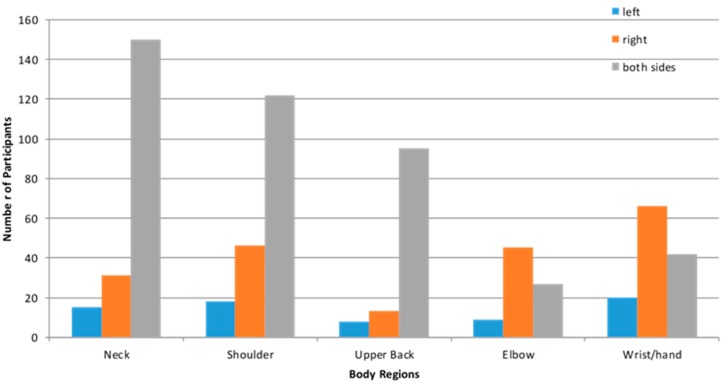
12 months prevalence of musculoskeletal discomfort in different body regions, and frequencies of left, right and bilateral symptoms.

In Table 4, the results showed the neck and shoulder regions were the most common areas of musculoskeletal discomfort, while the average discomfort scores were fairly low. In terms of duration of discomfort, there was a widespread range from very recent to over six months. From Figure 1, it can be seen that a large number of participants reported bilateral discomforts in the neck, upper back and shoulder areas. For the distal forearm and wrist/hand discomforts, the right side is the most common.

### 3.3. Psychosocial Stress Factors

**Figure 2:**
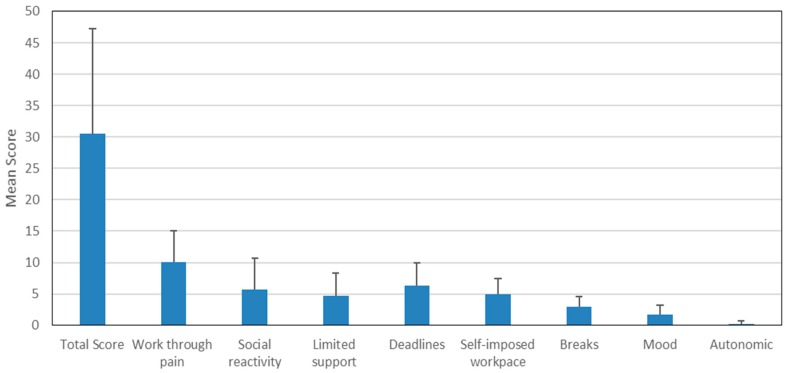
Workstyle total and subscale scores in participants (*n* = 285).

The total score from the Workstyle Short Form had a mean value of 30.05 (±16.82), and median score of 29.00 (±16.82) for the entire group. The total scores and the subscale scores are shown in Figure 2. Past studies have adopted the total score of 28 as a cut-off point for “adverse” workstyle [23], with 0–27 considered as “healthy” workstyle. In a cohort study of a large group of office workers in a Dutch government institute, it was reported that those with scores in the adverse workstyle range had a higher incidence of upper extremity discomforts even at 8 to 12 months follow-up [23,24].

### 3.4. Association of IT Use, Musculoskeletal Discomfort and Psychosocial Stress Factors

The findings of the logistic regression models are summarized in Table 5. The statistical significance and odd ratios are shown for those factors that reached a *p* value < 0.05. Odd ratios (OR) are the exponential functions of the regression coefficient associated with a one-unit increase in the exposure [25]. The OR values of greater than 1.0 suggests that the risk factor would lead to increased odds of outcome occurring. Results indicate that only laptop time and keypad phone time showed significant associations with neck pain but not with desktop or touchscreen phone time. The odds ratio (OR) for laptop time and keypad phone time actually showed values less than 1.0, suggesting that the use of these devices were associated with reduction in neck pain. Desktop time was found to be significantly associated with symptoms in the shoulder (OR = 1.094, 95% CI = 1.005–1.191), elbow as well as wrist/hand regions. The workstyle subscale of working through pain was the only factor that showed significant associations with each of the five body regions where symptoms were reported (Table 5).

## 4. Discussion

### 4.1. Cumulative Exposure to IT

The present results showed a mean value of 7.38 h ± 5.20 of daily use of the various types of devices. As this figure was computed by adding the self-reported data from the different categories, it may be considered a rough estimate of the actual use time. Berolo et al. [26] reported on the time spent with mobile devices among Canadian university students (average = 5.05 h per day), excluding computer use such as desktop or laptop. However, the types of handheld mobile devices used were not clearly defined in that study. Nevertheless, they have reported time spent in performing different types of tasks such as email, texting, internet browsing, making phone calls, gaming and so on. In the present study, the tasks performed using multi-touch devices were reported, and this area certainly needs more extensive investigations in the future. In view of the rising popularity of the multi-touch devices and increasing variety of tasks and applications that are emerging in the market, it is likely that the time spent on these new products would increase even more in the near future especially among the younger generations.

The finger actions involved in using these touchscreen devices may be very light, yet performing such actions at high speeds may still add to the cumulative loads of the musculoskeletal structures. There have been a few reports about excessive texting associated with development of arthritis in the first carpometacarpal joint [27], and tendonitis or tenosynovitis in the forearm and hand region [28,29]. Gold et al*.* [6] studied the postures of over 800 university students and reported that “a flexed neck and typing-side non-neutral wrist” were the most commonly observed posture during mobile device texting. These results are consistent with our current finding with over 80% of participants adopting a posture with the mobile device held at below eye level and without any forearm support. The tablet computer was associated equally (about 30% each) with resting the device on a table, on the laps or in mid-air; and these would still involve a flexed neck posture generally. Such a posture, if sustained for a long time, leads to increased risk of bilateral neck and shoulder pain.

In the present study, the neck region had the highest overall prevalence rates, followed by shoulder, wrist/hand, upper back and elbow among the participants. This is consistent with the recent research that has been published on the prevalence of musculoskeletal complaints associated with mobile device use [12]. Generally, the results on body regions suggest a bilateral pattern of discomforts in the spinal regions of the neck and upper back, while the wrist/hand and elbow discomforts were more common in the right side. The discomfort ratings reflect a mild level of intensity on the whole, and the durations of discomforts seem to suggest a rather recent pattern. This may be related to the age groups of the participants, with two-thirds in the age range of 18–35.

### 4.2. Relationship of IT Use, Workstyle and Musculoskeletal Discomforts

The total workstyle score was significantly correlated with total IT time, and it is most significantly correlated with daily desktop time. Desktop time also showed significant associations with the workstyle subscales of limited workplace support, meeting deadlines, self-imposed work pace and mood. Among the various IT devices, use of desktop computers is most likely during official working hours. Hence the participants may associate this factor with their workstyle. In contrast, the use of other IT devices such as smartphones and tablet computers may be more associated with leisure and entertainment, hence they were not significantly associated with increased psychosocial stress. It is not clear if there is a cause-effect relationship between IT use and psychosocial stress factors and to gain more insight into this relationship would require research of a prospective design. By understanding the psychological factors and behaviours of individuals who use mobile devices intensively, effective interventions or recommendations can be developed to address the health issues.

From the logistic regression models, the results generally suggest that desktop time showed a modest association with discomfort in shoulder and wrist/hand region. This is expected as long duration of computer use has been well documented to be a significant risk factor for work-related musculoskeletal disorders [5,16]. Sharan’s study [19] on a large cohort of Indian computer workers also found duration of computer use per day to be highly correlated with workstyle factors. Both of these factors were significant contributors to musculoskeletal discomfort as well as loss of productivity.

### 4.3. Use of Touchscreen Devices

In the present study, we did not find any significant association among touchscreen mobile device use and increased discomfort in any region. This was somewhat unexpected. It may be due to the demographic characteristics of the present cohort (greater number working vs. university students). In the study on Canadian university students by Berolo et al. [25], it was found that the “total” mobile devise time (over 2.375 h per day) contributed to increased risk for neck and shoulder pain with OR in the range of 2.01 (95% CI = 1.02–3.95) to 2.72 (95% CI = 1.24–5.96). It is envisaged that with the increased use of smartphone technology, daily exposure to these devices will be associated with a modest increase in reports of musculoskeletal discomfort among young adults. It is of interest to compare the cumulative exposure to IT use between different age groups in a larger study in the future.

### 4.4. Limitations of Study and Suggestions for Future Research

The present study only examined a cohort of 285 individuals using a convenience sampling method, and this sample size is not sufficient for identifying a significant relationship between electronic device use and discomfort. The smartphone and tablet technology is advancing at a very rapid pace, and new products and new applications are emerging very quickly on the market. It is possible that the self-reported daily exposure to these technologies may not be an accurate estimation of the real-world situation. Past research has suggested that self-reported data on computer interaction is often an over-estimation of the actual use time compared to data in observational studies [30,31]. Hence future studies should examine a larger population of individuals and compare the self-reported and observed data in using various devices. It is also possible to extract the actual user interaction data directly from the smartphone devices with the current technological advances. Direct observations of postures and input methods in using multi-touch devices may also reveal important information about the biomechanical loading involved. It is also important to study the effects of prolonged static postures in using these mobile devices.

## 5. Conclusions

The present study has reported on the cumulative exposures of IT use including smartphone and tablet technology among 285 adults in Hong Kong. Total IT time was significantly associated with the total workstyle score, although the duration of desktop use was still the most important factor associated with increased scores in the workstyle subscales. The workstyle subscale of “working through pain” showed significant associations with nearly all the different regions of musculoskeletal discomforts, and desktop time showed a significant association with shoulder and wrist/hand discomforts. Although the results did not reveal very strong associations of mobile device use with musculoskeletal discomfort, in view of the rising trend in smartphone use as a very popular tool in our daily lives, it is important to pursue further research on the effects of cumulative mobile device use affecting musculoskeletal health.

## Figures and Tables

**Table 1:** Demographic profile of participants (*n* = 285).

Demographic Factors	Whole Group	Males (*n* = 140)	Females (*n* = 145)
Age			
18–25	77	27	50
26–30	54	28	26
31–35	48	26	22
36–40	36	20	16
41–45	20	12	8
46–50	30	18	12
>50	15	8	7
missing	4	1	3
Body build			
Height (cm)	166.7 ± 8.9	173.2 ± 6.4 ^a^	160.4 ± 6.2 ^a^
Weight (kg)	61.4 ± 13.6	69.6 ± 12.4 ^b^	53.2 ± 9.3 ^b^
Work status (*n* = 278)			
Working full-time	221	117	104
Studying full-time	57	21	36
Others/Missing	7	2	5
Employment sector (*n* = 279)			
Banking	13	5	8
Food	9	6	3
Construction	11	9	2
Manufacturing	7	2	5
Education	18	11	7
Healthcare	71	27	44
Sales	14	8	6
Others	88	51	37
Missing	6	2	4
Handedness (*n* = 279)			
Left	20	13	7
Right	259	123	136
Missing	6	4	2
Sit in office all day			
Yes	151	70	81
Exercise regularly (*n =* 285)			
Never	14	4	10
Occasionally	154	55	99
1–2×/week	82	60	22
≥3×/week	31	19	12
Missing	4	2	2

^a,b^ Significant difference between groups at *p* < 0.05.

**Table 2:** Number of participants reporting daily use of various IT devices (*n* = 285).

	Total Number of Respondents (*n*, %)	<1 h	1–2 h	2–4 h	4–6 h	6–8 h	>8 h
Desktop Computer	225 (78.9%)	34 (11.9%)	38 (13.3%)	40 (14.0%)	36 (12.6%)	43 (15.1%)	34 (11.9)
Laptop Computer	146 (51.2%)	52 (18.2%)	44 (15.4%)	27 (9.5%)	15 (5.3%)	6 (2.1%)	2 (0.7%)
Mobile Phone (Keypad)	69 (24.2%)	42 (14.7%)	15 (5.3%)	5 (1.8%)	1 (0.4%)	4 (1.4%)	2 (0.7%)
Mobile Phone (Smartphones)	256 (89.8%)	50 (17.5%)	87 (30.5%)	54 (18.9%)	32 (11.2%)	13 (4.6%)	20 (7.0%)
Tablet Computers	89 (31.2%)	35 (12.3%)	32 (11.2%)	12 (4.2%)	6 (2.1%)	1 (0.4%)	3 (1.1%)
Handheld Electronic Game Devices	50 (17.5)	37 (13.0%)	9 (3.2%)	2 (0.7%)	0 (0%)	0 (0%)	2 (0.7%)

All percentages are calculated from the total number of participants (*n* = 285).

**Table 3:** Characteristics of touchscreen device use-duration, tasks and postural factors.

	Smartphones (*n* = 287)	Tablet Computers (*n* = 72)
How Long Since Used		
0–6 months	50 (17.4%) *	21 (29.2%) *
6–12 months	65 (22.6%)	24 (33.3%)
1–2 years	104 (36.2%)	22 (30.6%)
2–3 years	39 (13.6%)	5 (6.9%)
>3 years	29 (10.1%)	0 (0.0%)
Hours Used per Day		
0–1 h	52 (18.1%)	20 (27.8%)
1–2 h	102 (35.5%)	30 (41.7%)
2–4 h	96 (33.4%)	20 (27.8%)
>4 h	36 (12.5%)	2 (2.8%)
Where Used		
At work	108 (37.6%)	14 (19.4%)
At home	163 (56.8%)	54 (75.0%)
Public transport/car	217 (75.6%)	20 (27.8%)
Others (e.g. restaurant)	120 (41.8%)	17 (23.6%)
Tasks Performed		
Type/text	156 (54.4%)	18 (25.0%)
Check email	134(46.7%)	30 (41.7%)
Surf www	202 (70.4%)	54 (75.0%)
Graphic work	3 (1.0%)	1 (1.4%)
Play game	153 (53.3%)	40 (55.6%)
Photo	136 (47.4%)	4 (5.8%)
Movie/TV	89 (31.0%)	39 (54.2%)
Read book	125 (43.6%)	31 (43.1%)
Listen to music	130 (45.3%)	15 (20.8%)
Others	18 (6.3%)	2 (2.8%)
Viewing Angle		
At eye level	49 (17.1%)	21 (29.2%)
Below eye level	234 (81.5%)	49 (68.1%)
Above eye level	1 (0.0%)	2 (2.8%)
Forearm Position		
Rest on table	48 (16.7%)	20 (27.8%)
Rest on laps	36 (12.5%)	24 (33.3%)
Arms in mid-air	196 (68.3%)	22 (30.6%)
others	5 (1.7%)	6 (8.3%)
Input Style		
Right thumb	56 (19.5%)	6 (8.3%)
Right index finger	168 (58.5%)	49 (68.1%)
Left thumb	8 (2.7%)	0 (0.0%)
Left index finger	3 (1.0%)	3 (4.2%)
Both thumbs and index fingers	26 (9.1%)	6 (8.3%)
All fingers evenly	16 (5.6%)	8 (11.1%)

* Percentage values are computed using the *n* value for the type of devices as denominator.

**Table 4:** Musculoskeletal discomforts for different body regions.

Question	Neck	Shoulder	Upper Back	Elbow	Wrist/Hand
	(*n* = 206)	(*n* = 186)	(*n* = 123)	(*n* = 90)	(*n* = 132)
Discomfort Rating (0–10) (mean(sd))	3.09 ± 2.68	2.90 ± 2.77	1.76 ± 2.55	1.13 ± 2.15	1.65 ± 2.32
Duration of Discomfort in Last 12 Months					
1–7 Days	19	15	3	2	7
8–30 Days	14	3	6	0	2
>30 Days	16	12	6	2	4
1–3 Months	8	1	2	2	4
3–6 Months	4	3	0	0	3
>6 Months	15	13	8	7	3

**Table 5:** Summary of final multivariate logistic regression models for the five major body regions of musculoskeletal symptoms.

Body Region with Symptoms (Yes/No)	Factor	Significance (*p* Value)	Odds Ratio (OR)	95% CI (Lower to Upper Bound)
Neck	Laptop Time	0.009 *	0.819	0.705–0.951
	Phone (keypad) Time	0.027 *	0.742	0.569–0.967
	Phone (touchscreen) Time	0.078	0.915	0.830–1.010
	WS Subscale: Work Through Pain	0.001 *	1.108	1.045–1.174
	Constant	0.579	1.200	
Shoulder	Desktop Time	0.037 *	1.094	1.005–1.191
	Phone (Keypad) Time	0.042 *	0.770	0.598–0.991
	Phone (Touchscreen) Time	0.018 *	0.880	0.805–0.980
	WS Subscale: Work Through Pain	0.001 **	1.111	1.046–1.180
	WS Subscale: Mood	0.093	1.186	0.972–1.448
	Constant	0.033 *	0.464	
Upper Back	Phone (Keypad) Time	0.041 *	0.463	0.221–0.968
	WS Subscale: Work Through Pain	0.029 *	1.069	1.007–1.135
	WS Subscale: Self-Imposed Workpace	0.016 *	1.160	1.028–1.309
	constant	0.000 **	0.169	
Elbow	WS subscale: Work through pain	0.007 **	1.078	1.021–1.138
	Constant	0.000 **		
Wrist/hand	Desktop Time	0.015 *	1.103	1.019–1.193
	Laptop Time	0.015 *	0.808	0.681–0.960
	Phone (keypad) Time	0.059	0.735	0.534–1.012
	WS subscale: Work Through Pain	0.001 **	1.093	1.035–1.154
	Constant	0.000 **	0.290	

* *p* < 0.05, ** *p* < 0.01.
